# Identification of Ear Morphology Genes in Maize (*Zea mays* L.) Using Selective Sweeps and Association Mapping

**DOI:** 10.3389/fgene.2020.00747

**Published:** 2020-07-20

**Authors:** Ting Li, Jianzhou Qu, Xiaokang Tian, Yonghui Lao, Ningning Wei, Yahui Wang, Yinchuan Hao, Xinghua Zhang, Jiquan Xue, Shutu Xu

**Affiliations:** ^1^The Key Laboratory of Biology and Genetics Improvement of Maize in Arid Areas of the Northwest Region, Ministry of Agriculture, College of Agronomy, Northwest A&F University, Xianyang, China; ^2^The Maize Engineering Technology Research Centre of Shaanxi Province, Yangling, China

**Keywords:** population characteristics, selective sweeps, genome-wide association study, co-expression, ear morphology

## Abstract

The performance of maize hybrids largely depend on two parental inbred lines. Improving inbred lines using artificial selection is a key task in breeding programs. However, it is important to elucidate the effects of this selection on inbred lines. Altogether, 208 inbred lines from two maize heterosis groups, named Shaan A and Shaan B, were sequenced by the genotype-by-sequencing to detect genomic changes under selection pressures. In addition, we completed genome-wide association analysis in 121 inbred lines to identify candidate genes for ear morphology related traits. In a genome-wide selection scan, the inbred lines from Shaan A and Shaan B groups showed obvious population divergences and different selective signals distributed in 337 regions harboring 772 genes. Meanwhile, functional enrichment analysis showed those selected genes are mainly involved in regulating cell development. Interestingly, some ear morphology related traits showed significant differentiation between the inbred lines from the two heterosis groups. The genome-wide association analysis of ear morphology related traits showed that four associated genes were co-localized in the selected regions with high linkage disequilibrium. Our spatiotemporal pattern and gene interaction network results for the four genes further contribute to our understanding of the mechanisms behind ear and fruit length development. This study provides a novel insight into digging a candidate gene for complex traits using breeding materials. Our findings in relation to ear morphology will help accelerate future maize improvement.

## Introduction

Maize (*Zea mays* L. ssp. *mays*), one of the most widely grown crops in the world, plays an essential role in global food security. It has been suggested that maize was domesticated from teosinte (*Zea mays* L. ssp. *Parviglumis*) about 10,000 years ago in the Balsas River Basin of southwestern Mexico (Schnable et al., 2009; Xiao et al., 2017). Originally, the teosinte was a wild plant with approximately 5 to 12 kernels in the ear and long tassel branches (Doebley et al., 1995; Wang et al., 1999). The number of kernels in modern maize varieties is far larger than that of teosinte and the long branches have disappeared. There are sharp distinctions between modern maize and teosinte in terms of plant morphology structure.

The morphological architecture of maize underwent a striking transformation mainly owing to selection by early agriculturalists and the local environment. This domestication stage continued for a long time. The original landraces appeared during this long-term domestication process (Yamasaki et al., 2005). In recent years, to increase production and quality levels, modern breeders have made great improvements to maize materials. Recent short-term artificial improvements of maize have resulted in the improvement of combining ability, including specific combining abilities and others (Souza et al., 2010; Viana et al., 2013). Besides phenotypic change, selection during the domestication and improvement processes also brought about reduced genetic diversity in selected genes (Yamasaki et al., 2005). In maize, diversity analyses showed a sequence diversity decrease in the promoter region of *Teosinte branched1* (*tb1*) which affects long branches (Wang et al., 1999). Analysis of the genomic history of North American maize found that genetic separation and linkage disequilibrium increased with maize improvement (Van Heerwaarden et al., 2012). Selection transformed the genome and the shape of maize during domestication and improvement (Shi and Lai, 2015). Such changes in the genome and phenotype during the selection process provide an opportunity to study the influence of selected genes on agronomic traits.

A number of studies have explored the genetic variants of selected traits that arose during the selection process in various species. In soybeans, more than 800 differentiated regions, selected during the domestication process, were observed in the genome (Han et al., 2016). In chrysanthemum, about 550 genomic regions underwent selection amongst the different chrysanthemum types (Chong et al., 2017). In rice, a total of 200 regions with selective signatures were detected in two major *indica* subpopulations. A large number of genes with known functions in these selected regions are associated with crucial agronomic traits, particularly grain yield (Xie et al., 2015). In maize, there are 484 selected features arising from domestication and 695 from improvement (Hufford et al., 2012). In fact, the selected traits in different breeding populations are diverse during maize improvement. Thus, identifying the genetic variants underlying agronomic traits in different populations, which were developed during crop improvement, will help us understand more about selection effects and, in turn, lead to further crop improvements.

With the advent of new technologies and the exploitation of diverse analysis approaches, it is now possible to identify important genetic variants related to selected traits. Selective sweeps have been used to dissect selected regions in the above-mentioned studies. In general, the selection of a target trait is always accompanied by other agronomic traits due to the genetic hitch-hiking effect (Xu, 2018). The genome-wide association study (GWAS) has also been widely used to identify loci linked to target traits (Li et al., 2013; Chen et al., 2018). GWAS has a higher resolution to obtain casual genes, but some will be false positive. Hence, combining selective sweeps and GWAS is an efficient way to identify selected candidates in relation to specific traits. Furthermore, gene co-expression networks can be set up using gene expression data, which help associate genes of unknown function with biological processes and prioritize candidate select/target genes or discern transcriptional regulatory programs. This process has been carried out on data from multiple plants and has illuminated many key events during plant development (Sarkar et al., 2014; Huang et al., 2017; Wisecaver et al., 2017; Yu et al., 2017). Each approach has its unique advantages and disadvantages. Therefore, combining multiple analysis methods is likely to be an effective way of identifying the genetic mechanisms of selected traits.

Traits relating to ear architecture such as ear length (EL), fruit length (FL), setting rate (SR), and barren tip length (BTL) are essential for the improvement of grain yield during the breeding process. Two heterotic groups (Shaan A group and Shaan B group), with high heterosis between them, were established during 10-year breeding programs. Usually, inbreds from the Shaan A group act as the female population and those from the Shaan B group act as male. These sex differences have been utilized during selection to increase combining ability. In this study, we found that some traits arising from lines developed during long-term breeding selection from Shaan A and Shaan B groups showed significant differences, particularly in relation to EL, FL, BTL, and SR. This provides a good resource for the study of genetic variants in EL, FL, BTL, and SR. Here, we identified massive selective regions in the inbred lines from Shaan A and Shaan B groups, and we dissected the genetic mechanisms of EL, FL, BTL, and SR using this breeding population. In addition, we developed global gene co-expression networks and spatiotemporal-specific processes of genes in selected regions and genes governing significant agronomic traits. Our research revealed some candidate genes associated with ear development, which provides a valuable data resource for plant genetics research and breeding.

## Materials and Methods

### Plant Materials and SNP Genotyping

A total of 208 maize inbred lines (AM208) were collected including 54 inbred lines from the Shaan A group (A54) and 154 inbred lines from the Shaan B group (B154) (Supplementary Table S1). The Shaan A and Shaan B groups are derived from one basic population which was constructed using several excellent varieties. Then, the elite inbred Ye478 (Reid group) and HuangZaoSi (Tang SPT group) from China were used to pull the basic population into Shaan A group and Shaan B group, which were then improved over a period of approximately 10 years. Within the two heterosis groups, Shaan A and Shaan B play the role as female and male, respectively. The materials in the two groups were selected based on the number of harvest ears, grain weight per ear and seed rate (seed weight/ear weight) by planting in high density, low nitrogen and low irrigation conditions. New inbreds can be filtered by conducting a combining ability test which crosses more than two elite inbreds (including Zheng58 and Chang7-2). Two hundred and eight inbred lines were sampled at the 3-leaf stage and the DNA was extracted using a modified CTAB method (Murray and Thompson, 1980). The genotypes were determined using tGBS technology (Dara2bio; LLC, Ames, IA, United States) (Li et al., 2018). Overall, 48,432 SNPs were retained. Later, the 48,432 SNPs were inputted into Beagle version 4.1 analysis software (Browning and Browning, 2007, 2016) and filtered by a minor allele frequency (MAF) cutoff point of 5% using PLINK version 1.90 software (Purcell et al., 2007). A set of 32,306 high-quality SNPs (MAF ≥ 0.05) was retained for further analysis. In addition, 121 lines were selected from the AM208 population for construction of an association population (AM121), of which 26 lines (A26) belong to A54 and 95 (B95) belong to B154. Genotype data from the association population were filtered from the AM208 population genotype file resulting in 32,306 SNPs which were further screened using a MAF (≥0.05). Ultimately, 32,051 high-quality SNPs were applied to the GWAS.

### Population Structure and LD Analyses

For the AM208, an UPGMA tree with 1000 bootstraps was constructed using MEGA 7.0 software and a set of 32,306 high-quality SNPs (Kumar et al., 2016). Principal component analysis (PCA) was performed using the GCTA tool on the high-quality SNPs (Yang et al., 2011). The output of the GCTA tool was inputted into R software so as to graphically display the PCA results. Additionally, A54 and B154 were separated to compute the linkage disequilibrium (LD) decay distance of each group. To avoid the effect of sample size, 54 inbreds (B54 re-sample) were selected from B154 100 times at random. The LD decay distance was calculated using PopLDdecay software (Zhang et al., 2019). For AM121, a kinship matrix was created and a PCA was performed using TASSEL software version 5.0 (Bradbury et al., 2007) and 32,051 SNPs (MAF ≥ 0.05).

### Screening for Selective Regions and Genome-Wide Associations (GWAS)

Selective regions typically include two features, high differentiation and low diversity (Doebley et al., 2006). Therefore, the nucleotide diversity (π) ratios (A54/B154), the genetics differentiation (*Fst*) and the Tajima’s *D* statistic were calculated using a sliding-windows approach (100 kb non-overlapping sliding window) for A54 and B154 (Schmutz et al., 2014). We identified the low diversiry regions in A54 by the bottom 10% πA54/πB154 value and in B154 by the top 10% πA54/πB154 value. When a window is located on both the top 10% of the pool’s empirical distribution for *Fst* and the low diversity regions in A54 or B154, the window is considered to be a selective region in either the Shaan A or the Shaan B group.

Phenotypic data and 32,051 SNPs from AM121 were used to uncover the genetic architecture of the target traits using GWAS and a linear mixed model (P + K) in TASSEL v.5.0 software. The threshold was set to 1 × 10^–3^ based on analysis results. When the associated regions of the significant SNPs relating to the target trait overlapped with the selected regions, these associated regions were considered to be candidate regions and subsequent analysis was performed. In these regions, all genes were identified using the MaizeGDB database^1^. To compare selected genes with those from published data, all v4 selected gene IDs were converted to v3 gene IDs.

### Field Experiment Design and Phenotypic Data Collection

For the association population, AM121 individuals were planted in two different fields, which were distinguished using the codes E1 and E2, in Yangling in Shaanxi Province, China in 2017. The experiment consisted of two replicates, each with two rows. Each row was 5 m long and 0.6 m wide. The planting density was 67,500 plants/ha. When the maize was mature, according to single ear weight, ten ears were selected in each plot in the first replicate to measure ear weight (EW, g), and five ears were selected to measure ear row number (ERN), ear diameter (ED, cm), kernel number per row (KNR), ear length (EL, cm), fruit length (FL, cm), barren tip length (BTL, cm) and the compute setting rate (SR, the ratio of FL to EL). The mean value per material for each parameter was calculated. Descriptive statistics, ANOVAs, and Pearson correlation analyses were conducted using SPSS v.22 software (IBM crop. Armonk, NY, United States). Broad-sense heritability (*H*^2^) was calculated as follows:

$$H=\frac{\sigma_{g}}{\sigma_{g}+\frac{\sigma_{e}}{k}},$$

where σ_*g*_^2^ is the genetic variance, σ_ç_^2^ represents error variance, and k is the number of environments (Hallauer and Miranda, 1981).

### Gene Co-expression Network Analysis

A total of 78 maize B73 RNA-seq datasets from multiple tissues (seed, endosperm, embryo, leaf, ear, tassel, silk, cob, root, shoot, pollen, anther, and SAM) and developmental stages, reported and available in a public database^2^, were used to construct gene co-expression networks (Chen et al., 2014; Li et al., 2014). Gene co-expression network analysis was performed using the R package WGCNA version 1.63 (Langfelder and Horvath, 2008). In addition, β was optimized to six to achieve a scale-free topology. Hierarchical clustering was used to identify gene modules with a dynamic tree-cutting algorithm based on the dissimilarity of gene connectivity. A NetworkAnalyzer plugin available in Cytoscape was used to calculate relevant network parameters, such as the degree of connection (Assenov et al., 2008). Modules were visualized using Cytoscape version 3.6.1.

### Functional Annotation of Genes

To obtain more complete annotation information, all protein sequences of maize were mapped to Swiss-Prot/UniProt, Pfam, InterPro, Gene Ontology (GO) and the Kyoto Encyclopedia of Genes and Genomes (KEGG) databases by eggnog-mapper. For a given gene set, the R package clusterprofiler was used to visualize GO terms (Yu et al., 2012). A GO term was considered significantly enriched if the adjusted *p*-value was lower than 0.05. The pathway maps were obtained from the KEGG database (Kanehisa et al., 2017). The pathway enrichment analysis was performed using KOBAS version 2.0, and a Benjamini and Hochberg adjusted *p*-value of 0.05 was used as the cut-off criterion (Xie et al., 2011).

### Expression and Statistical Analysis

To avoid an infinite value, gene values expressed as zero were replaced with a value of 0.01. All of the samples were normalized using log_2_ (expression values + 0.01). Hierarchical clustering analysis was performed using the R package pheatmap^3^ and Pearson’s correlation co-efficient as the distance measure. Additionally, a Venn diagram was drawn using the VennDiagram package in R (Chen and Boutros, 2011).

## Results

### Genetic Divergence Between Inbred Lines From Shaan A and Shaan B Groups

**FIGURE 1:**
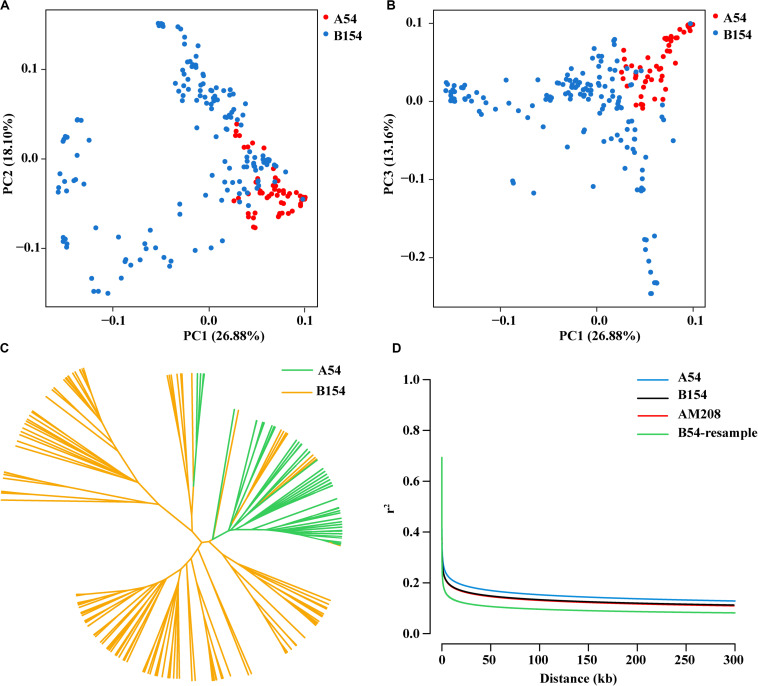
Population characteristic of AM208. **(A)** Scatter plot for PC1 and PC2. **(B)** Scatter plot for PC1 and PC3. **(C)** Phylogenetic tree of AM208 was constructed with 32,306 SNPs. **(D)** Linkage disequilibrium (LD) decay of AM208, A54, and B154.

In order to enlarge the germplasm resource of maize and screen elite inbred lines, the two heterosis groups (Shaan A group and Shaan B group) were built and improved. A series of inbred lines from both groups were selected for breeding and genetic study during improvement. To reveal genetic divergence between inbred lines from the Shaan A and Shaan B groups at the genomic level, principal component analysis (PCA) and an UPGMA tree were conducted using 32,306 high-quality SNPs with a MAF greater than 0.05. In the PCA, most of the inbred lines from the Shaan A group were separated from the Shaan B group lines using the first two or three eigenvectors (Figures 1A,B). Inbreds from Shaan B group were dispersed. In the UPGMA tree, except for several inbreds, inbred lines from the Shaan A group also clustered together, as did inbred lines from the Shaan B group (Figure 1C). This illustrated that Shaan A group and Shaan B group have occurred divergence in the genome, but some inbreds from the Shaan A and Shaan B groups are more closely related. Therefore, continuous genetic improvement in the future is needed to achieve further population divergence. Furthermore, in terms of the LD decay distance (*r*^2^ reached 0.2), AM208 was approximately 10 kb, and A54 had a longer LD decay distance even when we compared it to results of the same number of inbred lines when based on random sampling from B154. This indicates that B154 has a higher genetic diversity than A54 (Figure 1D).

**FIGURE 2:**
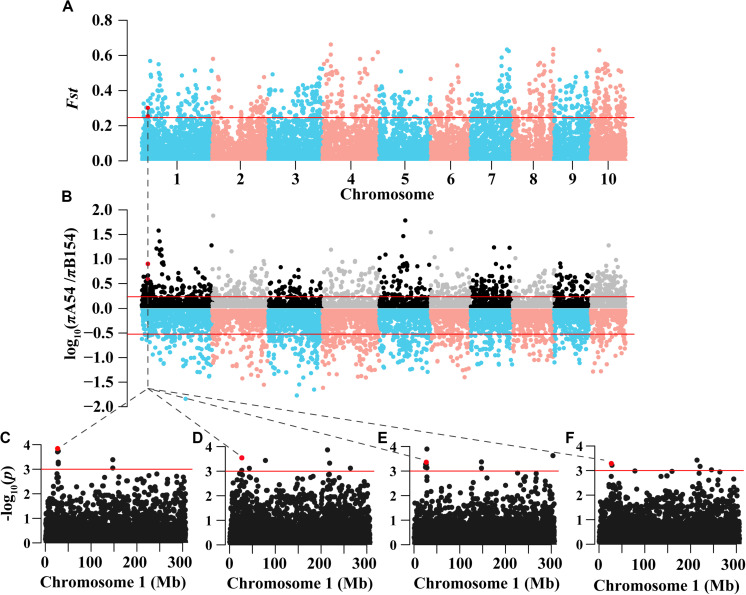
Genomic regions through long-term artificial selection in AM208 and Manhattan plot of EL and FL in Chromosome 1. **(A)** Distribution of genetics differentiation (*Fst*) values. **(B)** Distribution of nucleotide diversity (π) ratio (πA54/πB154). These values are calculated in 100kb sliding windows. Red lines represent the 90% tails of the empirical distribution of each stastic. **(C)** Region plot of EL in E1. **(D)** Region plot of EL in E2. **(E)** Region plot of FL in E1. **(F)** Region plot of FL in E2.

To identify the selective regions, the *Fst* and π ratio values between A54 and B154 were calculated using a 100kb step length. A54 had a lower nucleotide diversity (median, πA54/πB154 = 0.854) than B154. In addition, 91 selected regions (9.1 Mb) with both low diversity and high differentiation were discovered in A54, which included 199 protein-coding genes. However, in B154, a total of 246 (24.6 Mb) selected regions were identified, which contained 573 protein-coding genes (Figures 2A,B and Supplementary Table S2). The number of genomic regions with selective sweep signals in B154 was approximately 2.7 times that of A54. Moreover, selective regions in A54 focus on chromosome 3 and 7 (48, 52.75%), but are generally distributed on 10 chromosomes in B154. In addition, of these selective regions, 15 in A54 and 38 in B154 have no genes.

### Overview of the Functional Diversity of Genes and the Co-expression Network in Selected Regions

To investigate the functional genes located in selected regions, GO analysis was performed. There were distinctive differences in the greatly enriched GO terms (*p* < 0.05) of selected genes between A54 and B154. Significantly, genes in the selected regions for B154 showed greater diversity in biological functions than those in A54, such as in hormone synthesis, cell development, and partial secondary metabolites (Supplementary Table S3). In addition, the selective regions located on specific chromosomes and the biological functional diversity of genes in different groups indicate that there is a chasm-like split in some inbred line phenotypes from the Shaan A and Shaan B groups.

**FIGURE 3:**
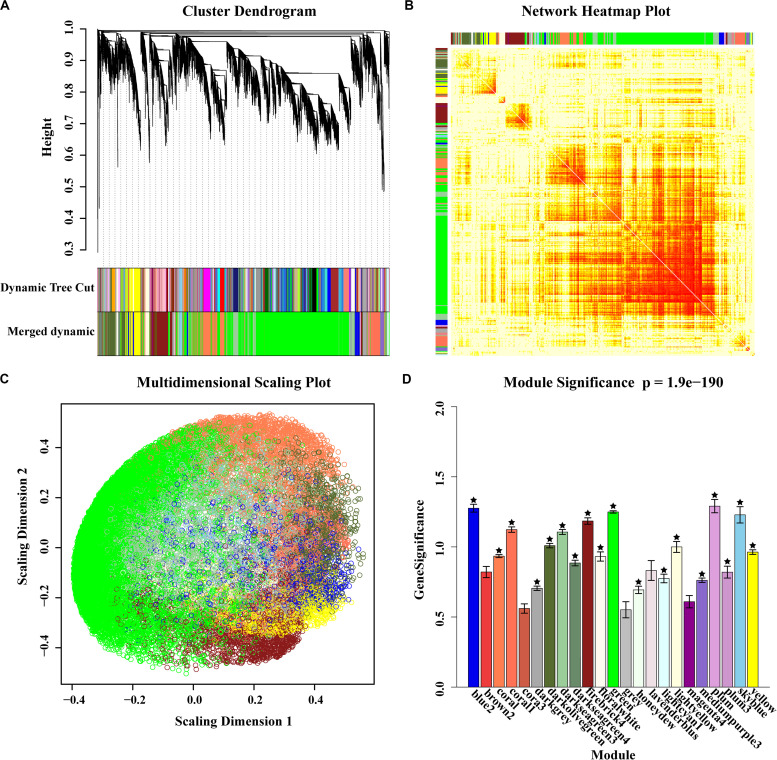
Gene modules identified by weighted gene co-expression network analysis (WGCNA). **(A)** Gene dendrogram obtained by clustering the dissimilarity based on consensus Topological Overlap with the corresponding module colors indicated by the color row. Each colored row represents a color-coded module which contains a group of highly connected genes. A total of 23 modules were identified. **(B)** Heatmap plot of topological overlap in the gene network. In the heatmap, each row and column corresponds to a gene, light color denotes low topological overlap, and progressively darker red denotes higher topological overlap. Darker squares along the diagonal correspond to modules. The gene dendrogram and module assignment are shown along the left and top. **(C)** A multi-dimensional scaling plot of genes indicates that the green module is the most distinct. **(D)** Bar plot of mean gene significance across modules, and the star tagged module represents where the genes of selected regions are distributed.

**FIGURE 4:**
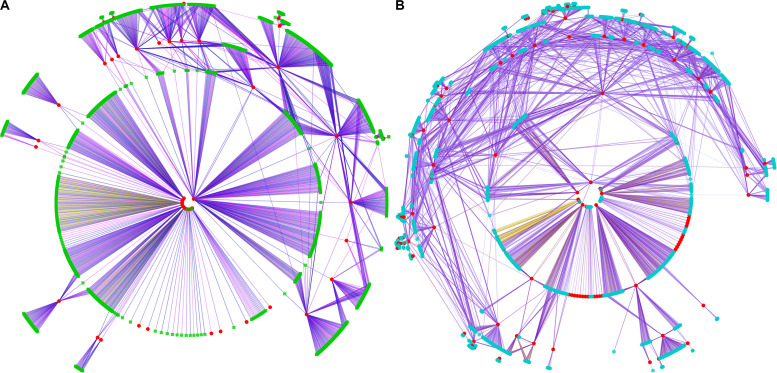
Co-expression network of genes in selected regions of A54 and B154. Graphical view of the co-expression network where the nodes correspond to genes and the edges to co-expression links. Different colored edges represent interaction modules of different selection genes. **(A)** Co-expression network of selected genes of A54, and selected genes marked in red, interactive genes were marked in green. **(B)** Co-expression network of selected genes of B154, and selected genes marked in red, interactive genes were marked in blue.

To gain a deeper understanding of the effect of genes in the selected regions, a weighted gene co-expression network analysis (WGCNA) was performed using gene expression data from multiple tissue types and developmental stages, which is available from a public database (see text footnote 2). A total of 23 WGCNA modules were identified in the analysis (Figure 3A). Of these, the green module was the most distinct (Figures 3B,C). Notably, genes in the selected regions of A54 and B154 were mainly distributed in 18 modules except for genes not included in the WGCNA (Figure 3D). To make it easier to follow the relationships among genes in selected regions and other unselected genes, 199 genes from A54 and 573 genes from B154, in selected regions, were screened as potential co-expression genes based on a weight value higher than 0.6. A co-expression network was then constructed (Figure 4). In total, 1086 genes were found to interact with selected genes from A54 and B154. In addition, 452 genes interacted with selected genes from A54 and 1315 genes interacted with selected genes from B154 (Supplementary Table S4). GO enrichment analysis indicated that the common interactive genes from A54 and B154 were mainly enriched during tissue development, regulation from vegetative to reproductive states, and pollen germination. Furthermore, remarkable functional differences were noted between specific genes in the selected regions of A54 and B154 (Supplementary Figure S1 and Supplementary Table S5). These results not only show new non-described gene associations and allow the placement in a functional context of some unknown non-assigned genes based on their interactions with known gene families, but also show that improvement for maize may has further accelerated the polarization of A54 and B154 in changing certain maize characteristics (such as quality and resistance) to satisfy breeding requirements.

### Genomic Association Analysis for Ear Morphology Components

**FIGURE 5:**
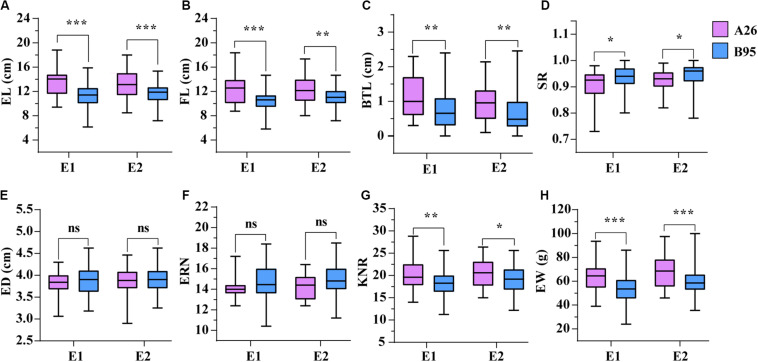
The comparison of eight traits between A26 and B95 in two environments. **(A)** Ear length (EL, cm). **(B)** Fruit length (FL, cm). **(C)** Barren tip length (BTL, cm). **(D)** Setting rate (SR). **(E)** Ear diameter (ED, cm). **(F)** Ear row number (ERN). **(G)** Kernel number per row (KNR). **(H)** Ear weight (EW, g). *, **, and *** indicate significant level at *P* < 0.05, *P* < 0.01 and *P* < 0.001, respectively. ns represents no significant difference.

Following functional enrichment analysis of the interactive genes, we found that these genes were mainly enriched during tissue development processes. During inbred selection, breeders give full attention to morphology and genetic diversity changes due to their importance for hybridization. Herein, we chose eight ear related traits containing ear length (EL), fruit length (FL), ear weight (EW), ear row number (ERN), ear diameter (ED), kernel number per row (KNR), barren tip length (BTL) and setting rate (SR) in order to observe phenotypic differences between 26 inbred lines (A26) from the Shaan A group and 95 inbred lines (B95) from the Shaan B group. Except for ERN and ED, there were significant differences in EL, FL, EW, SR, BTL, and KNR between A26 and B95 in the two environments (Figure 5). In addition, EL was significant positively correlated with FL (*r* = 0.95). However, SR and BTL were significant negatively correlated (Supplementary Figure S2). These results indicate that ear related traits were significantly altered in the Shaan A and Shaan B groups during population improvement process.

**FIGURE 6:**
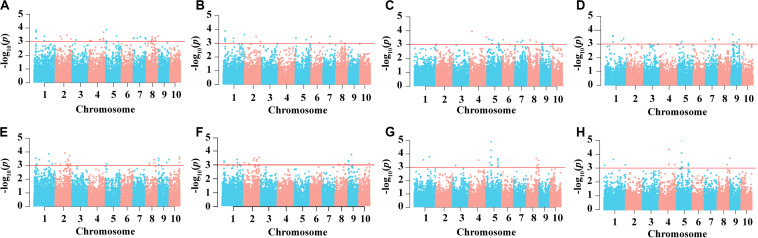
Result of genome-wide association study. Manhattan plots of EL in E1 **(A)** and in E2 **(E)**, FL **(B)** and in E2 **(F)**, BTL **(C)** and in E2 **(G)** and SR in E1 **(D)** and in E2 **(H)**.

In the AM121 population, the broad-sense heritability (*H*^2^) of the eight ear traits were higher than 60%, ranging from 65.33% for BTL to 79.09% for EW (Table 1). Considering the observed phenotypic differences between A26 and B95 and the correlation coefficient between traits, EL, FL, BTL and SR were chosen to help conduct the GWAS with 32,051 high-quality SNPs, analyzed using a linear mixed model (P + K). As shown in Supplementary Figure S3, the probability of a false positive result has been reduced in this study. In total, 84, 49, 47, and 50 significant SNPs were identified for EL, FL, SR, and BTL, respectively (Figure 6 and Supplementary Table S6). Among these SNPs, 5, 4, 3, and 0 SNPs in relation to EL, FL, BTL and SR were co-localized in two locations, respectively. Furthermore, we noticed that the majority of significant SNPs were co-localized between EL and FL, as were BTL and SR. Especially, three co-localized SNPs associated with EL and FL located at chromosome 1 were observed with the same *p*-value due to the high LD (*r*^2^ = 1.0) (Figures 2C–F). In relation to these three significant SNPs, two different haplotypes (GGG, AAA) were identified. Haplotype 2 (AAA) had a longer EL and FL. Approximately 95.79% (91) of B95 was associated with Haplotype 1 (GGG) and 38.46% (10) of A26 was associated with Haplotype 2 (AAA) (Supplementary Table S7). In addition, none of co-localized significant SNPs were identified in relation to BTL and SR in both environments. The reason for this may be that BTL and SR have a lower heritability than EL and FL. According to the LD decay distance reported in previous research, the upstream and downstream 150 kb of the significant SNPs are regarded as associated regions (Li et al., 2018). Associated regions including the same genes were considered as one associated region for each trait. Finally, 404, 182, 207, 154 protein-coding genes (reference maize genome v4) distributed in these associated regions were found to be associated with EL, FL, BTL, and SR, respectively (Supplementary Table S6).

**TABLE 1:** The basic statistics of ear length (EL), fruit length (FL), barren tip length (BTL) and setting rate (SR), ear weight (EW), ear diameter (ED), ear row number (ERN), and kernel number per row (KNR) of AM121 in two environments.

Trait	Environment	Range	Mean ± SD	SV (%)	*H*^2^ (%)
EL (cm)	E1	6.13–18.80	11.79 ± 2.03	17.22	67.50
	E2	7.18–18.00	11.95 ± 1.88	15.73	
FL (cm)	E1	5.80–18.36	10.92 ± 1.92	17.58	69.84
	E2	7.18–17.34	11.23 ± 1.81	16.12	
BTL (cm)	E1	0.00–2.40	0.83 ± 0.58	69.88	65.33
	E2	0.00–2.46	0.71 ± 0.53	74.65	
SR	E1	0.73–1.00	0.93 ± 0.05	5.38	65.60
	E2	0.78–1.00	0.94 ± 0.04	4.26	
ED (cm)	E1	3.06–4.62	3.86 ± 0.32	8.29	75.18
	E2	2.90–4.62	3.90 ± 0.30	7.69	
ERN	E1	10.40–18.40	14.48 ± 1.61	11.12	66.75
	E2	11.20–18.50	14.82 ± 1.47	9.92	
KNR	E1	11.25–28.80	18.46 ± 3.25	17.61	69.39
	E2	12.20–26.40	19.36 ± 3.20	16.53	
EW (g)	E1	24.00–93.50	55.34 ± 12.43	22.46	79.09
	E2	35.50–100.00	61.07 ± 11.99	19.63	

### Gene Co-location: Combining Selective Sweeps and Association Analysis

By integrating selective sweeps and GWAS, we identified 5 regions related to EL or FL and another 5 associated regions related to BTL or SR overlapping with the selected regions (Table 2). There was a difference between the size of the selected window and the associated region. Therefore, the 10 associated regions were designated as candidate regions so as to avoid missing relevant genes. Interestingly, among these regions, the candidate region (26,738,827–27,038,887 bp), located on chromosome 1, was from the three co-localized SNPs related to EL and FL (above-mentioned) (Figures 2C–F). According to information from the reference genome sequence (www.maizegdb.org_v4), four protein-coding genes were found to be distributed in this 300 kb candidate region, which contains *Zm00001d028216*, *Zm00001d028217*, *Zm00001d028218*, and *Zm00001d028219*.

**TABLE 2:** Co-localized regions detected by combining selective sweeps and GWAS.

Chr	Selected region	log_10_ (π ratio)	*Fst*	Associated region	Trait	Environment	*P*-value
1	26700001–26800000	0.902	0.301	26738827–27038887	EL, FL	E1, E2	5.16E-04
1	26800001–26900000	0.592	0.254	26738827–27038887	EL, FL	E1, E2	5.16E-04
1	303500001–303600000	0.315	0.512	303442010–303742023	FL	E1	2.36E-04
1	78400001–78500000	1.098	0.469	78287346–78587346	EL	E2	3.66E-04
1	78500001–78600000	1.179	0.491	78287346–78587346	EL	E2	3.66E-04
4	220900001–221000000	0.260	0.278	220783246–221083246	SR	E2	5.57E-04
4	238600001–238700000	0.387	0.285	238349228–238649228	BTL	E1	2.88E-04
4	32800001–32900000	0.710	0.498	32741698–33041698	BTL	E1	1.10E-04
6	121600001–121700000	0.648	0.449	121310639–121610639	FL	E2	9.37E-04
7	36700001–36800000	-1.132	0.328	36536622–36836622	BTL, SR	E1	6.12E-04
9	24000001–24100000	0.468	0.313	23836153–24136153	BTL	E1	8.00E-04
10	147000001–147100000	0.544	0.270	146955309–147393999	EL, FL	E2	7.16E-04
10	147100001–147200000	0.535	0.355	146955309–147393999	EL, FL	E2	7.16E-04

Of the four candidate genes, *Zm00001d028216* was annotated as *indeterminate floral apex1* and synonyms with a C2C2-YABBY transcription factor, which has been found to be involved in regulating the determinacy of the floral meristem, spikelet pair meristems and spikelet meristems (Laudenciachingcuanco and Hake, 2002). The second gene, *Zm00001d028217*, was annotated as the developmental protein SEPALLATA 2 and descripted as a MADS transcription factor*-MADS14*. It has a wide range of functions, such as regulating flowering time, vegetative development and fruit ripening, especially in the meristem, and is related to floral organ identity (Cacharr et al., 1999; Heuer et al., 2001; Setter et al., 2001; Danilevskaya et al., 2008; Thompson and Hake, 2009; Zhang et al., 2012). The other two genes may have essential functions in defining the boundary of secondary walls and are described as follows: *Zm00001d028218* encodes a methionine-tRNA ligase, which catalyzes a reversible chemical reaction [from ATP, L-methionine and tRNA (Met) to AMP, diphosphate and L-methionyl-tRNA (Met)] in cytosol; and *Zm00001d028219* encodes a microtubule-associated protein (MAP70-2), which is closely related to MAP70-1 (Calder, 2010; Korolev et al., 2010; Oda and Fukuda, 2012). These results further indicate that the four genes we observed in our study are likely to play roles in the regulation of EL and FL, particularly in relation to the complex genetic mechanisms of EL and FL.

### Temporal and Spatial Expression Patterns and a Co-expression Network of Key Genes Associated With EL and FL

**FIGURE 7:**
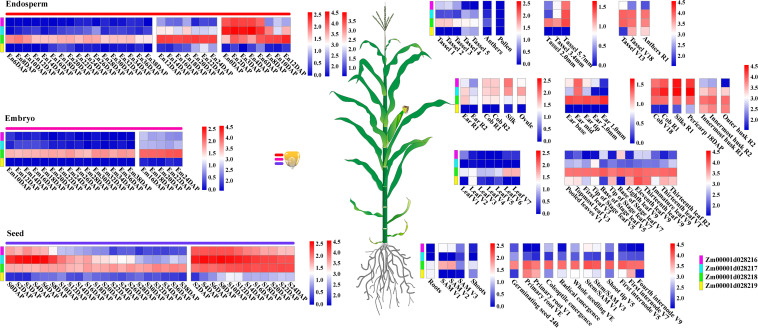
Temporal and spatial expression patterns of regulation genes of EL and FL. Different expression modules in same tissue represent different expression data sources, and the scale bar shows the normalized expression values corresponded to different modules. Additionally, different colored bars represent four different genes.

To explore and characterize the expression patterns of four target genes of EL and FL in a complex tissue, public RNA-seq datasets were used to analyze temporal and spatial expression variations of these genes in a large and diverse group of maize B73 tissues. Interestingly, *Zm00001d028216*, *Zm00001d028217*, and *Zm00001d028219* were expressed in specific tissues or developmental stages. However, the expression of *Zm00001d028218* was not remarkably different between any developmental stages, yet its expression level was higher than the three other genes in almost all tissues (Figure 7). *Zm00001d028216* and *Zm00001d028217* exhibited synchronous expression patterns in some specific developmental stages or tissues. For example, they were highly expressed in the early developmental stages of endosperm, seed, mid-ear, tassel (5.7 mm), silk, and pericarp, and especially in the cob. In particular, both *Zm00001d028216* and *Zm00001d028217* showed higher expression levels in the specific developmental stages of parts of the ear, silk, and ovule (Figure 7). These expression results indicate that *Zm00001d028216* and *Zm00001d028217* are likely to be key regulators during the determination of the meristem and the later differentiation and development of the ear. The synchronization and similarity in the expression characteristics of these genes also suggest that they coordinate and regulate the development of certain tissues, but the hub genes between them remain unknown.

**FIGURE 8:**
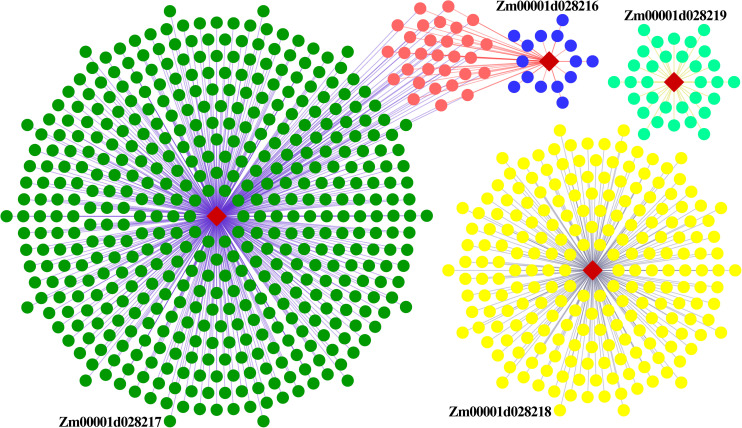
Regulatory co-expression network of key genes related to EL and FL. Weighted gene co-expression gene network of multiple maize tissues, visualized using an organic layout in cytoscape. The nodes in the network were obtained by coloring each gene by its specific expression profile along maize tissues development. Different genes and their interactive genes were marked with different color. Of which, blue nodes represent weight value more than 0.5, orange nodes represent common interactive genes of *Zm00001d028216* and *Zm00001d028217*.

To gain further insight into the relationship between these genes, and the influence of external genes, genes that potentially interact with them were extracted from the co-expression network to construct sub-networks (based on a weight value greater than 0.2). The most important finding of the co-expression network was that some hub genes were involved in sub-networks that had *Zm00001d028216* and *Zm00001d028217* at the core (Figure 8). These hub genes were composed of 28 protein-coding genes, and included three transcription factors. They were mainly involved in cell differentiation, cellular developmental processes, and anatomical structure morphogenesis (Supplementary Table S8). Nevertheless, the other two genes constituted a relatively independent network, and they were functionally enriched in a number of critical developmental processes. This indicates that EL and FL are affected by diverse biological processes, and multilevel genes may be involved in communicating and adjusting the function balance among these hub genes.

## Discussion

During crop improvement, phenotypic characteristic developments are dependent on market demand and breeder selection. Micro-evolution during the breeding process not only increases genetic divergence, but also changes genetic diversity. In this study, 32,306 high-quality SNPs were used to identify the effect of artificial improvement across the genome, and our analysis suggests that there has been genetic divergence between the Shaan A and B groups in AM208. These results indicate that artificial selection was effective in causing population divergence between Shaan A and Shaan B heterotic groups. This is consistent with previous findings that selection in breeding programs makes significant contributions to genetic divergence between heterotic groups (Liu et al., 2003). Moreover, B154 is more dispersed and has a shorter LD decay distance than A54. Also, the median π ratio (πA54/πB154 is equal to 0.854) further supports these differences. Our results also indicate that B154 contains a richer genetic diversity than A54. Additionally, it is particularly interesting that B154, which has a richer genetic diversity, has more selection regions. Our analysis provides new and important information on the breeding history genomics of Shaan A and Shaan B groups.

Furthermore, a subset of 337 regions including 772 genes in the genome that are related to artificial selection were identified. Within the 772 selected genes, approximately 24 candidate genes including *Zm00001d028217* have been reported in a previous study to be associated with modern breeding selection (Jiao et al., 2012). In addition, 20 including *Zm00001d028216* and 22 candidate genes overlapped with candidate genes related to maize domestication and improvement, respectively (Hufford et al., 2012; Supplementary Table S9). These combined results indicate that these genes are of high value for crop improvement. A large proportion of identified genes have not been reported because they are from different populations, are related to different focus traits from different breeders, or are identified using different genotyping technology. We found that there are distinctive differences in gene numbers in selected regions between A54 and B154. Enrichment analysis revealed that these genes are involved in regulating different essential metabolic processes during maize development (due to the different usage of their materials during the breeding process). Moreover, the co-expression networks captured important biological modules of selected genes between A54 and B154. These modules showed that these genes were not likely to be independently regulating these metabolic processes, and were also not the only key factors in causing the divergence in some target traits. Additionally, the selected genes and the interactive genes were only a little enriched in response to abiotic stress. This is likely to be because these inbreds were selected under the same conditions: low irrigation rates, low nitrogen levels, and high plant densities. The interactive genes with selected genes were enriched in the regulation of the timing of meristematic phase transition and transition from the vegetative to reproductive phase, as well as pollen germination. These are important targets for a female and male plant, implying that genotype and phenotype difference may be generate gradually during improvement of heterosis groups. Taken together, our results indicate that artificial selection alters consciously or unconsciously the alleles’ frequencies of target traits during improvement process.

In the maize breeding process, construction of heterosis groups is considered to be the most important element. Hybrid vigor remarkably contributes to the performance of hybrids compared to their parents (Birchler et al., 2003). Therefore, parents’ inbred lines may have obvious complementation in the trait and genome. EL, FL, BTL, and SR are vital to increasing yield. In this study, A54 performed longer EL and FL than B154. However, B154 showed higher SR (equivalent to shorter BTL) than A54. The combination crossing from these two groups may produce hybrids with long ears and high SR, such as the SD650 (KA105/KB024) and SD636 (KA103/KB043) hybrids that have been widely grown in Shaanxi province. In this study, four candidate genes related to EL and FL were identified by combining GWAS and a selective sweep. Among the four genes, *Zm00001d028216* and *Zm00001d028217* also were identified as selected genes in maize domestication and improvement processes (Hufford et al., 2012; Jiao et al., 2012). Moreover, *Zm00001d028217* belongs to the MADS family and *Zm00001d028216* belongs to the YABBY family, these two transcription factors play important roles in regulating spikelet development, floral induction, and inflorescence development (Cacharr et al., 1999; Laudenciachingcuanco and Hake, 2002; Danilevskaya et al., 2008; Thompson and Hake, 2009). Alternatively, *Zm00001d028216* and *Zm00001d028217* showed higher expression levels at the development stage and are known to regulate other genes involved in cell differentiation, cellular developmental processes, and anatomical structure morphogenesis. Therefore, it is meaningful and interesting to carry out further validation work on these four genes to understand the underlying molecular biology mechanism. In addition, short BTL (higher SR) is an desirable trait for high-yield breeding, however, genetic basis of BTL and SR remain poorly understand (Li et al., 2020). In the present study, only five associated regions related to SR and BTL overlapped with the selected regions. Moreover, many significant SNPs associated with SR and BTL were identified through association analysis. Therefore, this study has helped to uncover the genetic mechanism of SR and BTL, information which can be used to aid genetic improvements. Nonetheless, some associated regions related to SR and BTL were detected, and more research is needed to identify the functional genes, particularly in the overlapped region.

Genetic analysis for complex traits generally requires genetic populations with a diverse phenotype. This may lead to a situation where researchers collect diverse materials from multiple breeding programs and geographical areas but do not focus on studying breeding materials from a single origin. We used 208 inbred lines selected from the Shaan A and Shaan B groups that were derived from a common ancestral pool but used different testers. As breeding populations, Shaan A and Shaan B groups integrate a diverse germplasm and allele frequency which have been subjected to noteworthy changes due to long-term artificial selection. More importantly, as homozygotes, inbred lines from Shaan A and Shaan B groups can be used as ideal test materials for genetic studies and marker-assisted breeding. In future, genetic studies that use breeding populations will greatly progress breeding applications. Our study used these inbreds from a breeding population to investigate genetic divergence and we identified the selective regions related to traits. This not only provides insights into the effects of artificial selection across the genome for crop improvement but also conveys essential information on some important agronomic traits.

## Data Availability Statement

The raw data supporting the conclusions of this article will be made available by the authors, without undue reservation, to any qualified researcher.

## Author Contributions

SX and JX conceived and designed the experiments. XZ, YH, and XT contributed to field management. YW, YL, and NW contributed to the collection of phenotypic data and DNA extraction. TL and JQ analyzed the data and wrote the manuscript. SX contributed to revising the manuscripts. All authors read and approved the final manuscript.

## Conflict of Interest

The authors declare that the research was conducted in the absence of any commercial or financial relationships that could be construed as a potential conflict of interest.

## Footnotes

1 https://www.maizegdb.org/gbrowse/maize_v4

2 https://maizegdb.org/

3 https://cran.r-project.org/web/packages/pheatmap/index.html

## Supplementary Material

FIGURE S1 GO analysis of interactive genes with selected genes in A54 and B154. Histogram showing the significantly functional distribution of interactive genes with selected genes in A54 and B154, respectively. The scale bar represents the significant levels.

FIGURE S2 Data distribution and correlation analysis of eight traits. ^∗^, ^∗∗^, and ^∗∗∗^ indicate a significant correlation at *P* < 0.05, *P* < 0.01, and *P* < 0.001 respectively.

FIGURE S3 QQ-plots of eight ear related traits in two locations. QQ-plot of EL in E1 **(A)** and E2 **(E)**. QQ-plot of FL in E1 **(B)** and E2 **(F)**. QQ-plot of BTL in E1 **(C)** and E2 **(G)**. QQ-plot of SR in E1 **(D)** and E2 **(H)**.

TABLE S1 The material information of AM208.

TABLE S2 The list of selective regions and genes in A54 and B154, respectively.

TABLE S3 GO enrichment analysis of selected genes from Shaan A and B groups.

TABLE S4 Interaction between selected genes and other genes.

TABLE S5 GO enrichment analysis of interactive genes of selected genes.

TABLE S6 Results of genome-wide association study for EL, FL, BTL, and SR in two environments.

TABLE S7 Haplotype effect of three significant SNPs related to EL and FL.

TABLE S8 Functional enrichment analysis of key genes regulating EL and FL.

TABLE S9 Common selected genes in maize domestication and improvement processes compared to previous studies.
