# Supplementary figures and images for: Identification of Ear Morphology Genes in Maize (Zea mays L.) Using Selective Sweeps and Association Mapping
Source: Front Genet. 2020 Jul 20;11:747. doi: 10.3389/fgene.2020.00747 (PMC7384441; doi:10.3389/fgene.2020.00747)

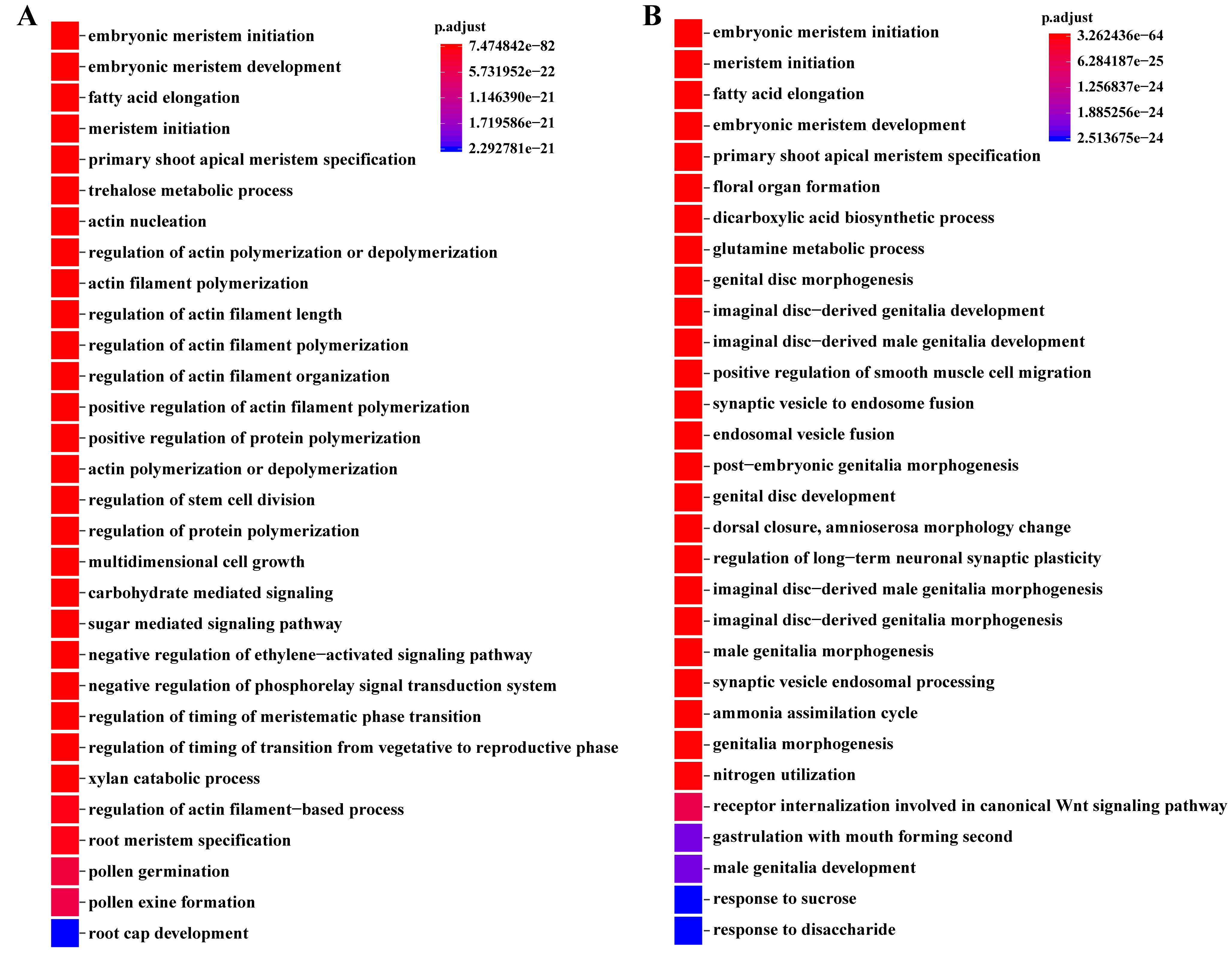

Supplement: FIGURE S1 — GO analysis of interactive genes with selected genes in A54 and B154. Histogram showing the significantly functional distribution of interactive genes with selected genes in A54 and B154, respectively. The scale bar represents the significant levels. [file Image_1.TIF]

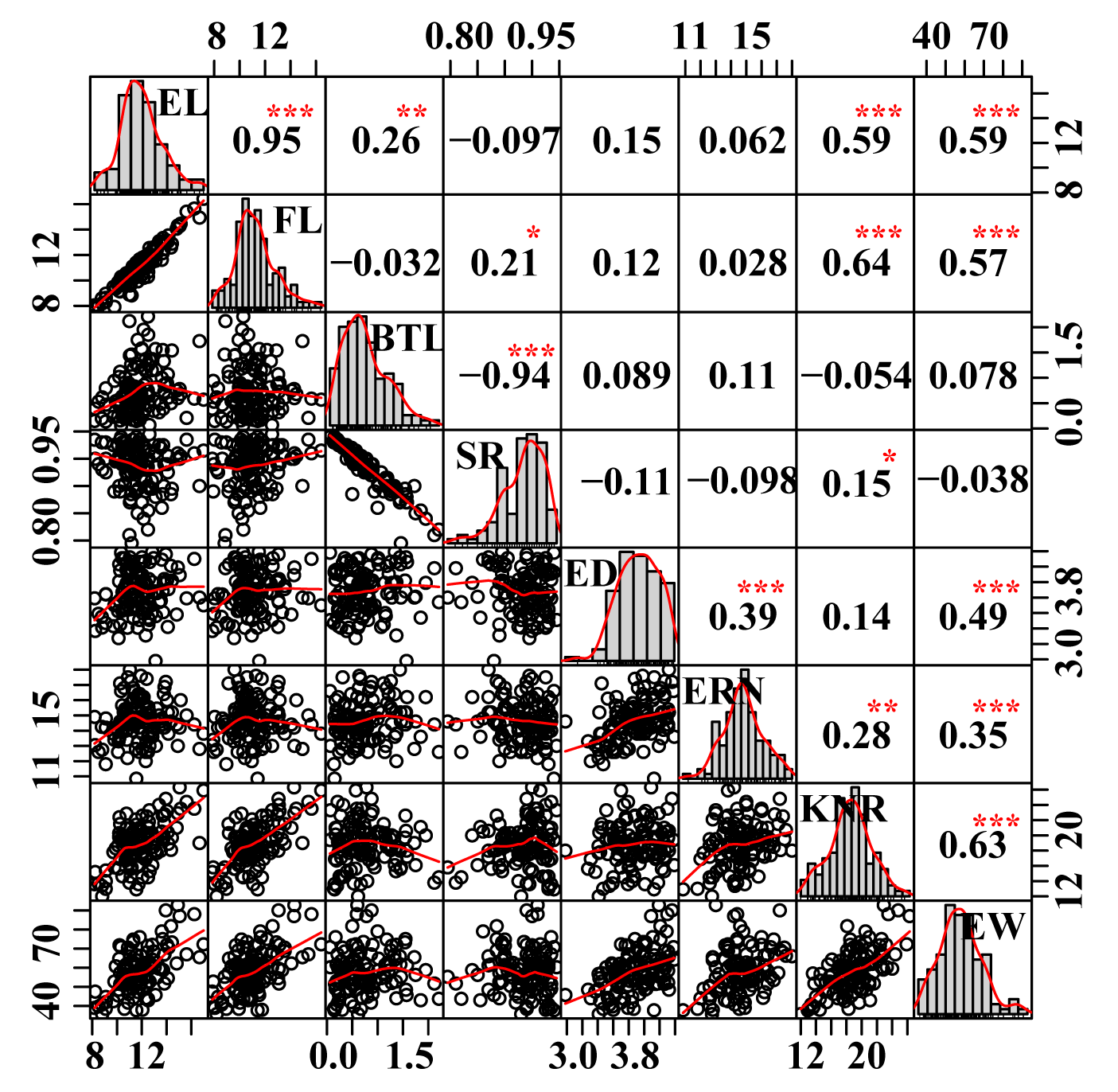

Supplement: FIGURE S2 — Data distribution and correlation analysis of eight traits. ∗, ∗∗, and ∗∗∗ indicate a significant correlation at P < 0.05, P < 0.01, and P < 0.001 respectively. [file Image_2.TIF]

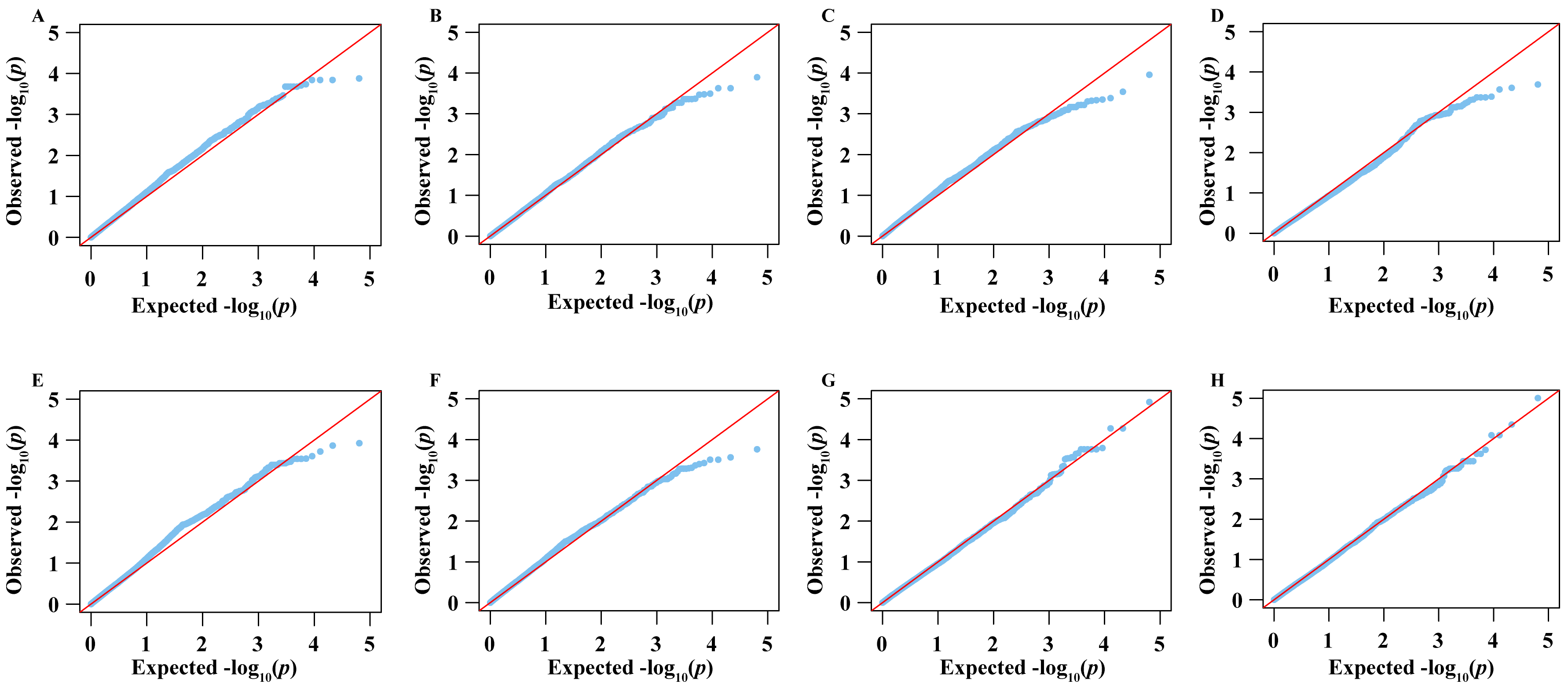

Supplement: FIGURE S3 — QQ-plots of eight ear related traits in two locations. QQ-plot of EL in E1 (A) and E2 (E). QQ-plot of FL in E1 (B) and E2 (F). QQ-plot of BTL in E1 (C) and E2 (G). QQ-plot of SR in E1 (D) and E2 (H). [file Image_3.TIF]
